# itol.toolkit accelerates working with iTOL (Interactive Tree of Life) by an automated generation of annotation files

**DOI:** 10.1093/bioinformatics/btad339

**Published:** 2023-05-24

**Authors:** Tong Zhou, Kuidong Xu, Feng Zhao, Weiyue Liu, Longzhao Li, Zhongyi Hua, Xin Zhou

**Affiliations:** Laboratory of Marine Organism Taxonomy and Phylogeny, Qingdao Key Laboratory of Marine Biodiversity and Conservation, Institute of Oceanology, Chinese Academy of Sciences, Qingdao 266071, China; Shandong Province Key Laboratory of Experimental Marine Biology, Institute of Oceanology, Chinese Academy of Sciences, Qingdao 266071, China; University of Chinese Academy of Sciences, Beijing 100049, China; Laboratory of Marine Organism Taxonomy and Phylogeny, Qingdao Key Laboratory of Marine Biodiversity and Conservation, Institute of Oceanology, Chinese Academy of Sciences, Qingdao 266071, China; Shandong Province Key Laboratory of Experimental Marine Biology, Institute of Oceanology, Chinese Academy of Sciences, Qingdao 266071, China; University of Chinese Academy of Sciences, Beijing 100049, China; Laboratory of Marine Organism Taxonomy and Phylogeny, Qingdao Key Laboratory of Marine Biodiversity and Conservation, Institute of Oceanology, Chinese Academy of Sciences, Qingdao 266071, China; Shandong Province Key Laboratory of Experimental Marine Biology, Institute of Oceanology, Chinese Academy of Sciences, Qingdao 266071, China; University of Chinese Academy of Sciences, Beijing 100049, China; Laboratory of Marine Organism Taxonomy and Phylogeny, Qingdao Key Laboratory of Marine Biodiversity and Conservation, Institute of Oceanology, Chinese Academy of Sciences, Qingdao 266071, China; University of Chinese Academy of Sciences, Beijing 100049, China; Laboratory of Marine Organism Taxonomy and Phylogeny, Qingdao Key Laboratory of Marine Biodiversity and Conservation, Institute of Oceanology, Chinese Academy of Sciences, Qingdao 266071, China; University of Chinese Academy of Sciences, Beijing 100049, China; National Resource Center for Chinese Materia Medica, China Academy of Chinese Medical Sciences, Beijing 100700, China; State Key Laboratory of Mycology, Institute of Microbiology, Chinese Academy of Sciences, Beijing 100101, China

## Abstract

**Summary:**

iTOL is a powerful and comprehensive phylogenetic tree visualization engine. However, adjusting to new templates can be time-consuming, especially when many templates are available. We developed an R package namely itol.toolkit to help users generate all 23 types of annotation files in iTOL. This R package also provides an all-in-one data structure to store data and themes, accelerating the step from metadata to annotation files of iTOL visualizations through automatic workflows.

**Availability and implementation:**

The manual and source code are available at https://github.com/TongZhou2017/itol.toolkit

**Figure:**
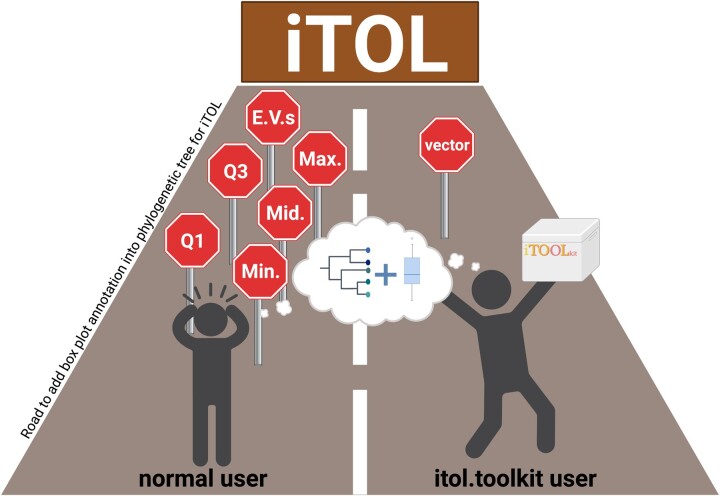
Two roads to add box plot annotation into a phylogenetic tree for iTOL.

## 1 Introduction

Reproducible visualizations require available data and code and knowing exactly how all parameter settings were used (Nekrutenko and Taylor 2012, Fekete and Freire 2020). Interactive visualization platforms are a double-edged sword that makes manipulation easier but workflow less reproducible. iTOL (Interactive Tree of Life, https://itol.embl.de/) is a powerful graphic engine for phylogenetic tree visualization and annotation (Letunic and Bork 2007) with more than 70 000 users (Letunic and Bork 2021). Compared to other feature-rich and programming-based tools, such as ggtree (Xu *et al.* 2022), phytools (Revell 2012), and baltic (Dudas *et al.* 2015), iTOL has a more user-friendly operating system, which only requires uploading annotation files to the iTOL website. But with the increasing number of annotation types, the learning curve of getting used to my different templates is rather steep. The lack of developer communities makes the challenge exist for a long time. Some third-party unpackaged scripts (Supplementary Table S1) that help users to generate annotation files in various template formats are available, and have been widely used for automatic analysis pipelines (Liu *et al.* 2021) or research (e.g. Korf *et al.* 2019, Xue *et al.* 2022, Hillary *et al.* 2022). However, they only support a part of template types (Supplementary Table S1). There is still a need of a helper tool for iTOL, just like ggtreeExtra for ggtree (Xu *et al.* 2021), to make the whole workflow easier and the iTOL functionally expanded.

**Figure 1:**
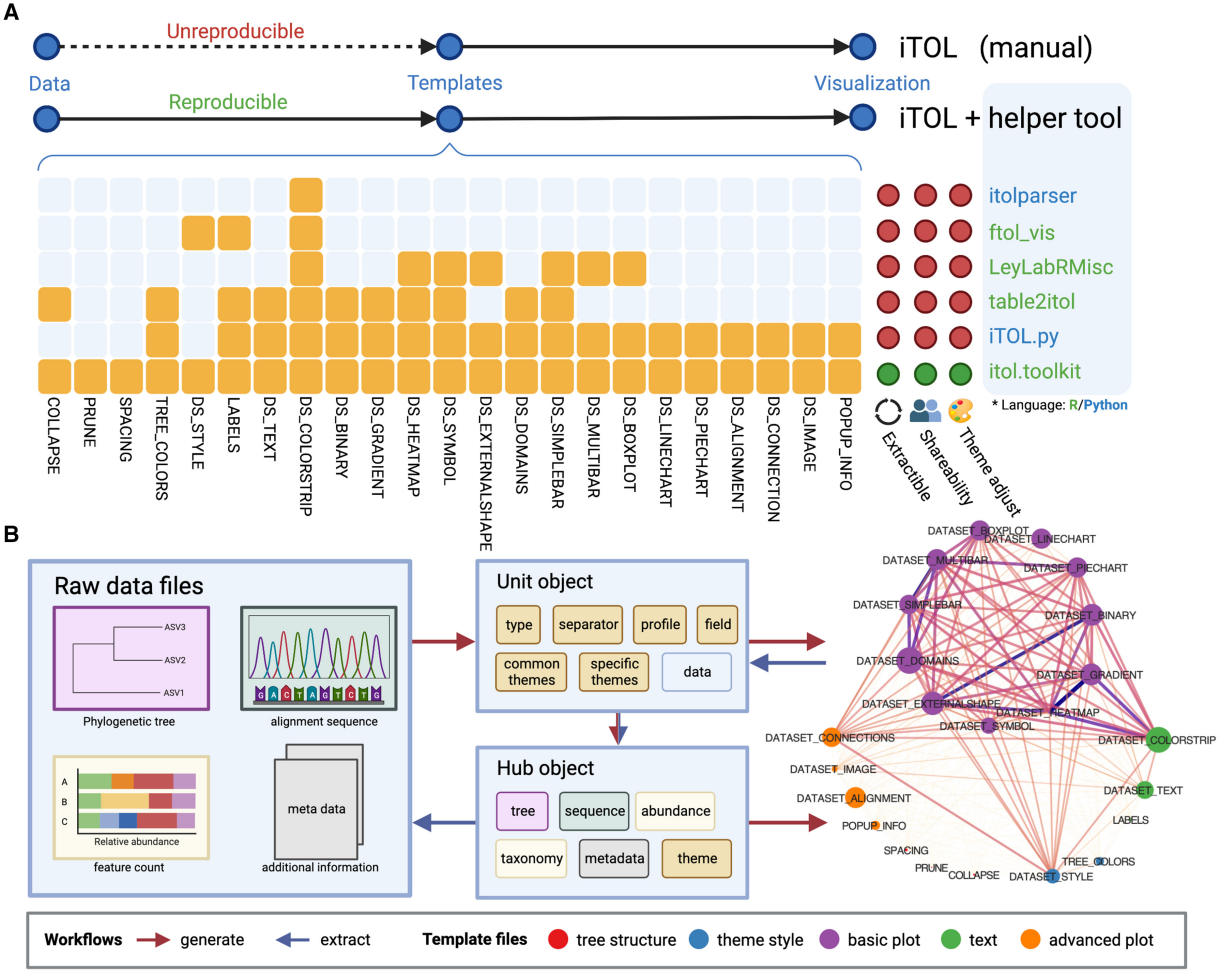
Helper tools comparison and main workflows of itol.toolkit package. (A) Supporting template coverage comparison among helper tools. (B) The generation and extraction workflows of itol.toolkit package. The similarity relationship between the template files is calculated by shared parameter count.

After comparing all parameters among the templates in iTOL (Supplementary Table S2), we found that the helper tools available failed to support generating all annotation types and the current iTOL lacks the function of reproducibility from data to template file (Fig. 1A), especially for the free version. Here, we provide the following shortcomings in iTOL and solutions in the developed open-source R package namely itol.toolkit for free version users:

1. Low-frequency specific parameters account for 71.05% (Supplementary Fig. S1), which is hard for users to learn, as the alignment template has 14 specific parameters only used once (Supplementary Table S2). We aggregated the parameters and hid the complex type-based operation in the background to smooth the learning curve even if iTOL releases more template types in the future.
2. The unusual input data format requires additional statistical calculation directly leading to a low usage rate (Supplementary Fig. S1). For example, the boxplot template needs a minimum, first-quarter, middle, third-quarter, maximum, and even unequal-length extreme values, not just a simple vector of numbers. In itol.toolkit, users only need to input a vector of numbers, and the program will automatically generate a formatting annotation file for iTOL.
3. Some powerful functions contain too much programming background to prepare data. For example, the text annotation supports HTML, which is more flexible for editing in a programming environment than the spreadsheet-based editor. We developed an interactive Shiny add-in in itol.toolkit to help users generate flexible text annotations without coding.

By overcoming these problems, itol.toolkit will greatly accelerate the work with iTOL by providing support for all types of iTOL annotations.

## 2 Features

### 2.1 Generate template files by simplified workflow

For most R operations, such as the input for the grammar of graphics in the ggplot2 package, the long format table is comfortable for R users. For the itol.toolkit user, generating a box plot template is as simple as the workflow in ggplot2. First, we prepared a minimum working example dataset (https://tongzhou2017.github.io/itol.toolkit/articles/Datasets.html#dataset-1) in the package for users to learn the workflow. The users can download the dataset and start from a local TXT or CSV format file as an input read by fread function in data.table package or use system.file function to get the file path in the package, as described in the quick start tutorial (https://tongzhou2017.github.io/itol.toolkit/articles/Get_Start.html). The first column of the table should be *x*-axis values, and the second column should be the *y*-axis values, as shown in the #9 layer of Supplementary Fig. S1. Second, the program automatically counts each descriptive statistic value (e.g. min and max) and the unequal-length vectors of extreme values. With the itol.toolkit helper tools, users do not need to calculate the values by themselves and prepare the template file manually (Fig. 1A).

For the line chart, the program can also automatically convert the long format table data into the template format. For the tree color, style, and color strip templates, the create_unit function automatically assigns color palettes according to the template type when there is no user definition, as shown in the #1, #4, and #7 layers of Supplementary Fig. S1. For text templates, the complex_html_text function (a Shiny add-in in RStudio, the GUI is shown in Supplementary Fig. S2) can be used to edit the HTML text just by clicking and selecting the parameters of color, size, font, and sub/sup position.

### 2.2 Extract data from template files

We used the extraction workflow to extract the default theme parameter in the iTOL official example template files as the built-in theme. The built-in theme helps users quickly generate template files without considering the details of the many theme parameters. Users who need a more flexible definition of theme can edit the unit object or theme slot of hub object directly. The theme adjusting flexibility is also a key difference between itol.toolkit and other helper tools (Fig. 1A). The popular help tool table2itol can generate annotation files, but users may find it difficult to change the color or other themes flexibly (Supplementary Table S5).

By extracting the template files of published papers, we found that the most used schemes are the same in the color palette as those in table2itol (https://github.com/mgoeker/table2itol), RColorBrewer, and ggsci packages. Hence, we depend on these packages to help users to generate the most popular color palettes. Users can use the color parameter of create_unit function to set up or change 55 schemes. The maximum length of schemes is up to 76, while it is only 40 in table2itol.

### 2.3 Shareable and reproducible result

The all-in-one S4 object stores data and themes. Hence, saving the hub object as an RData file is shareable between different computers with the same version of itol.toolkit. The write_hub function supports to extract data for which the theme name is the same as the prefix of the data name. It achieves high-throughput reproduction from hub object to template files in seconds (Fig. 1B). This reproducible data object is more suitable than separated code and data by other helper tools to be submitted to public reproducible materials hubs (Fig. 1A).

## 3 Usage and documentation

itol.toolkit is hosted on GitHub (https://github.com/TongZhou2017/itol.toolkit) and a detailed user manual (https://tongzhou2017.github.io/itol.toolkit/) is available.

## Supplementary Material

btad339_Supplementary_Data

## Data Availability

All data are incorporated into the article and its online supplementary material.
