# Supplementary figures and images for: itol.toolkit accelerates working with iTOL (Interactive Tree of Life) by an automated generation of annotation files
Source: Bioinformatics. 2023 May 24;39(6):btad339. doi: 10.1093/bioinformatics/btad339 (PMC10243930; doi:10.1093/bioinformatics/btad339)

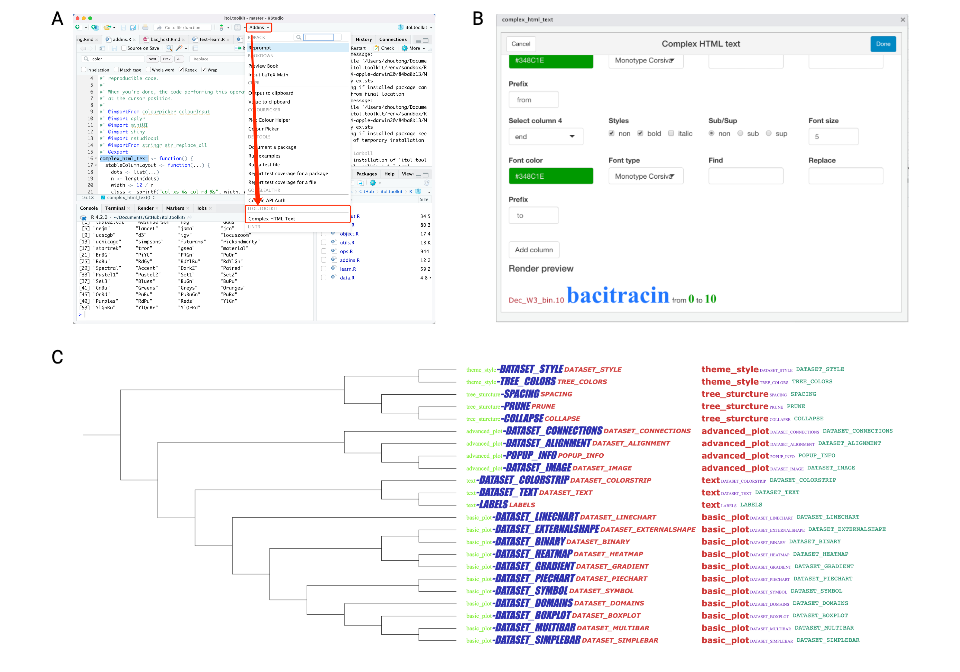

Supplement: btad339_Supplementary_Data [file btad339_supplementary_data.zip › Figure S2 HTML text RStudio add-in GUI.png]

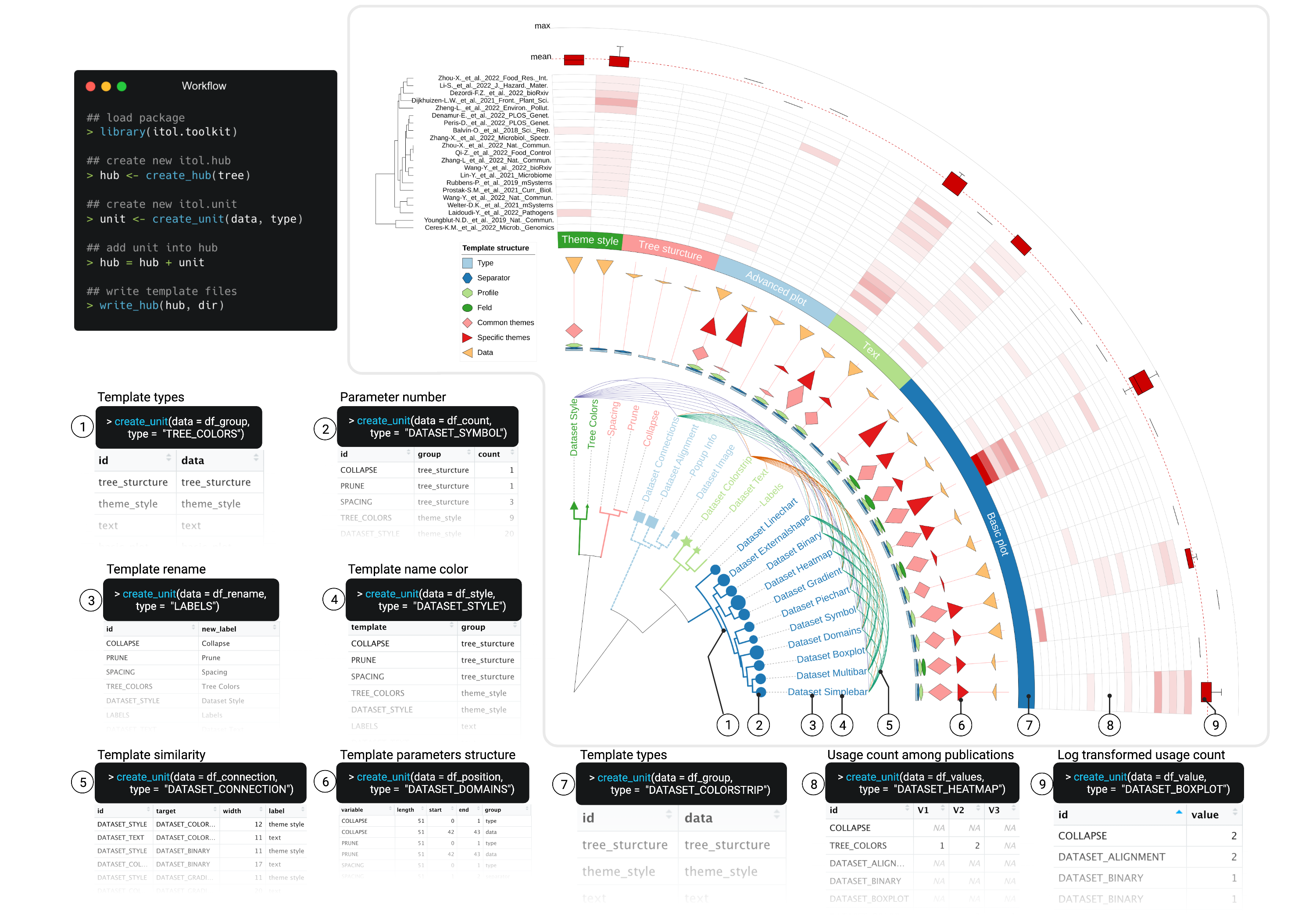

Supplement: btad339_Supplementary_Data [file btad339_supplementary_data.zip › Figure S1 Overview of template types usage of iTOL and itol.toolkit R command.png]
